# Hemoglobin and Its Z Score Reference Intervals in Febrile Children: A Cohort Study of 98,572 Febrile Children

**DOI:** 10.3390/children10081402

**Published:** 2023-08-17

**Authors:** Chu-Yin Cheng, Ting-Hsuan Hsu, Ya-Ling Yang, Ying-Hsien Huang

**Affiliations:** 1 Department of Emergency Medicine, Kaohsiung Chang Gung Memorial Hospital, Kaohsiung 833, Taiwan; 2 Department of Anesthesiology, Kaohsiung Chang Gung Memorial Hospital, College of Medicine, Chang Gung University, Kaohsiung 333, Taiwan; 3 Department of Pediatrics, Kaohsiung Chang Gung Memorial Hospital, College of Medicine, Chang Gung University, Kaohsiung 333, Taiwan

**Keywords:** hemoglobin, Z scores of Hb, CRP, children, bacteremia, age, Kawasaki disease

## Abstract

Objectives: Febrile disease and age of children were associated with a variation in hemoglobin (Hb) level. Both CRP and Hb serve as laboratory markers that offer valuable insights into a patient’s health, particularly in relation to inflammation and specific medical conditions. Although a direct correlation between CRP and Hb levels is not established, the relationship between these markers has garnered academic attention and investigation. This study aimed to determine updated reference ranges for Hb levels for age and investigated its correlation with CRP in febrile children under the age of 18. Methods: This is a cohort study of in Chang Gung Memorial Hospitals conducted from January 2010 to December 2019. Blood samples were collected from 98,572 febrile children who were or had been admitted in the pediatric emergency department. The parameters of individuals were presented as the mean ± standard deviation or 2.5th and 97.5th percentiles. We also determined the variation of Hb and Z score of Hb between CRP levels in febrile children. Result: We observed that the Hb levels were the highest immediately after birth and subsequently underwent a rapid decline, reaching their lowest point at around 1–2 months of age, and followed by a steady increment in Hb levels throughout childhood and adolescence. In addition, there was a significant and wide variation in Hb levels during the infant period. It revealed a significant association between higher CRP levels and lower Hb levels or a more negative Z score of Hb across all age subgroups. Moreover, in patients with bacteremia, CRP levels were higher, Hb concentrations were lower, and Z scores of Hb were also lower compared to the non-bacteremia group. Furthermore, the bacteremia group exhibited a more substantial negative correlation between CRP levels and a Z score of Hb (r = −0.41, *p* < 0.001) compared to the non-bacteremia group (r = −0.115, *p* < 0.049). Conclusion: The study findings revealed that the Hb references varied depending on the age of the children and their CRP levels. In addition, we established new reference values for Hb and its Z scores and explore their relationship with CRP. It provides valuable insights into the Hb status and its potential association with inflammation in febrile pediatric patients.

## 1. Introduction

Childhood anemia is a widespread health concern impacting young children [1,2] and the hemoglobin (Hb) reference varies according to the children’s age, especially in the infant period [3]. Several studies have established detailed age- and sex-specific reference intervals for hematologic parameters in healthy children [4,5]. It is worth noting that these reference ranges can exhibit slight variations depending on factors such as the number of cases considered, and the specific age intervals examined. Moreover, generating Hb reference data on a large cohort from infant or childhood populations presents ethical and practical challenges. Febrile illnesses are the most frequently reported primary concern among children seeking medical attention in pediatric emergency departments and a significant proportion of cases, ranging from 10% to 25%, involving febrile illness are attributed to bacterial infections [6,7]. Currently, clinicians will use reliable history-taking, physical examination findings, and standard laboratory investigations that can aid in distinguishing between benign viral infections, cases of over-swaddling, and potentially serious bacterial infections in febrile children [8].

As an acute-phase reactant synthesized by the liver in response to inflammation, C-reactive protein (CRP) levels in the blood can rapidly increase in reaction to various inflammatory processes within the body [9,10]. CRP is a biomarker most commonly used in the evaluation of febrile children to assess the presence and severity of inflammation [11,12]. It is important to note that CRP levels alone cannot diagnose a specific condition but are used in conjunction with other clinical assessments and laboratory tests to help determine the cause of fever and guide appropriate management [13,14]. Elevated CRP levels indicate the activation of the acute-phase response, which is typically associated with infectious or inflammatory conditions [15]. Monitoring CRP levels can aid healthcare professionals in evaluating the necessity for additional diagnostic tests, making informed treatment choices, and gauging the effectiveness of therapy in pediatric patients with fever [15,16,17]. Starting from February 2020, the globe has been immersed in a rigorous battle against the COVID-19 illness, leading to immense strain on health systems as the ailment evolved into a pandemic. Of note, elevated CRP levels can facilitate the early detection of oxidant–antioxidant levels and necessitate specialized attention for individuals in critical condition with COVID-19 [18]. Meanwhile, CRP stands out as one of the highly efficient biomarkers for predicting mortality risk associated with COVID-19 [18,19,20,21,22].

The World Health Organization (WHO) classifies anemia in children under 5 years of age as a condition where the Hb concentration is below 11 g/dL without arbitrary references [3]. Anemia or decreased Hb levels can be observed in febrile children and may indicate various underlying causes, including infectious or inflammatory conditions [23,24]. These low Hb levels are a non-specific indicator that warrants further investigation to identify the precise cause, enabling appropriate diagnosis and treatment. In the presence of infection, inflammatory cytokines can stimulate the production of hepcidin in the liver [25,26]. This leads to an increase in macrophage activation and the destruction of red blood cells, while simultaneously suppressing erythropoiesis [26]. As a result, anemia associated with inflammation can occur due to a combination of hepcidin-induced hypoferremia and cytokine-mediated inhibition of erythropoiesis, leading to a shortened lifespan of erythrocytes [23,26,27]. Monitoring Hb levels in febrile children can provide valuable insights into the severity of the illness and guide appropriate management strategies [7,24,28]. We have also observed lower Hb and higher CRP levels in children with bacteremia [7], urinary tract infection [29], or Kawasaki disease in our previous studies [24,28]. The combination of Hb and CRP can be an effective diagnostic approach for these cases [24,30].

The primary aim of this study was to establish updated reference ranges for Hb levels according to age and investigate their correlation with CRP in febrile children under 18 years old. This was a retrospective study and we enrolled febrile children who presented to PER of memorial hospitals in Chang Gung and had laboratory blood tests from January 2010 to December 2019 during this ten-year study period. We proposed the Hb references according to age, utilizing a real-world large dataset and providing a standardized Z score of Hb as an adjunct index for assessing Hb level in the febrile pediatric population with CRP levels.

## 2. Materials and Methods

### 2.1. Study Population

**Figure 1:**
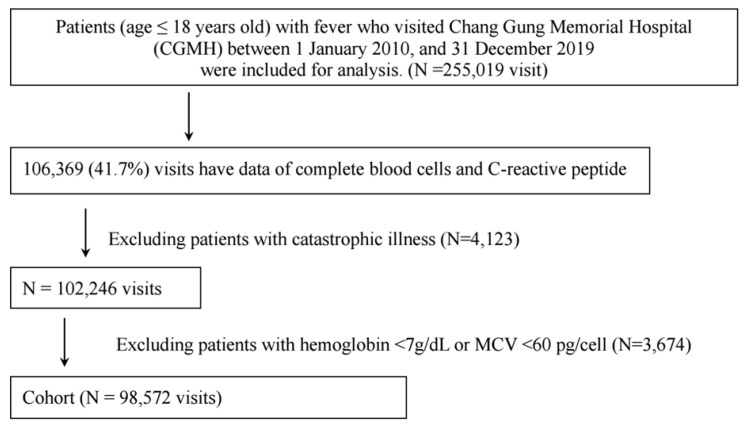
The figure illustrates the methodology used in this study. We evaluated febrile children at four major branch hospitals of the Chung Gung Medical Foundation in Taiwan. The research cohort comprised 255,019 visits by individuals below the age of 18 from 2010 to 2019. The research cohort comprised 255,019 visits by individuals below the age of 18 from 2010 to 2019. Out of a total of 106,369 febrile children (41.7%), laboratory tests were administered. After excluding patients with catastrophic illness (N = 4123) and those with Hb levels below 7 g/dL or MCV < 60 pg/cell (N = 3674), a final count of 98,572 eligible patients remained in the cohort for subsequent analysis.

For enrollment in this study, we evaluated febrile children at four major branch hospitals of the Chung Gung Medical Foundation in Taiwan. These hospitals include two medical centers located in Linkou and Kaohsiung, as well as two regional hospitals in Keelung and Chiayi. The Chang Gung Memorial Hospital (CGMH) network stands as a remarkable achievement, holding the esteemed position of being Taiwan’s most expansive and all-encompassing healthcare service provider. With an impressive capacity of over 10,000 beds, CGMH exemplifies a commitment to comprehensive medical care that is unparalleled in the region [27]. Its share encompasses more than a tenth (1/10) of the medical services provided in Taiwan. The study period for enrollment spanned from January 2010 to December 2019. This study was granted through the formal approval obtained from the Institutional Review Board (IRB) of the CGMH (#202201305A3). Demographic characteristics (age and gender) and laboratory values, including Hb levels, and CRP data were collected. We excluded febrile children who had a catastrophic illness, Hb less than 7 g/dL, or a mean corpuscular volume (MCV) of 60 pg/cell. The flowchart of this study is displayed in Figure 1. In order to comprehensively account for potential age-related discrepancies, the febrile participants were meticulously categorized into a total of thirteen distinct groups. This meticulous categorization encompassed a broad spectrum of age intervals: ranging from the earliest age cohort spanning from 0 to 8 days, gradually progressing through various developmental phases up to the age of 18 years. The resulting age groups included the following intervals: 0–8 days, 8 days–1 month, 1–2 months, 2–4 months, 4–6 months, 6–12 months, 1–2 years, 2–4 years, 4–7 years, 7–10 years, 10–11 years, 11–12 years, and finally, 12–18 years. Furthermore, within this meticulously structured age framework, a secondary division was implemented to address the potential fluctuations in CRP levels within specific age ranges. This entailed a partitioning of participants into four distinct groups, each characterized by differing CRP levels: <5 mg/L, 5–30 mg/L, 30–100 mg/L, and >100 mg/L. This dual-stratification strategy enabled a comprehensive exploration of age-related variations in conjunction with CRP levels, facilitating a deeper comprehension of the interplay between these critical variables.

### 2.2. Laboratory Study

The blood samples obtained were promptly analyzed at the hospital laboratories within a time frame of 0.5 h after sampling. Various parameters include complete blood cell and CRP levels. Additionally, the MCV and mean corpuscular Hb concentration were calculated using the instrument. We calculated the Z scores of Hb values and compared the characteristics of these values to the absolute Hb within the group with CRP less than 5 mg/L. This analysis allowed us to assess the relationship between Z scores of Hb and absolute Hb concentrations, considering the specific characteristics of each group. The formula of Z score = ((measure value − mean value)/standard deviation).

### 2.3. Blood Culture

Among the microorganisms isolated from the blood samples, certain pathogens were identified, indicating their role as true pathogens. These included Staphylococcus aureus, Streptococcus pneumoniae, Salmonella enterica, group A streptococci, Pseudomonas aeruginosa, Hemophilus influenzae, Escherichia coli, Candida species, and other relevant organisms [7,31]. However, certain pathogens are more likely to represent contamination rather than true infection. These include coagulase-negative staphylococci, Staphylococcus epidermidis, Corynebacterium species, Gram-positive Bacillus, Micrococcus, and similar organisms [31,32]. Accurate diagnosis and proper treatment decisions rely on differentiating between true pathogens and contaminants. This differentiation is crucial in ensuring effective medical management. Our selection process for confirmed bacteremia cases is based on the aforementioned criteria.

### 2.4. Statistical Analysis

The experimental results were presented as mean ± deviation or 2.5th and 97.5th percentiles. To compare the two groups, a two-tailed Student’s *t*-test was employed in cases where the quantitative data exhibited a normal distribution. We used Pearson’s correlation coefficient method for two variables where the quantitative data exhibited a normal distribution. To evaluate the comparison of categorical data such as gender between groups, an analysis was conducted using either the chi-squared test or Fisher’s exact test. The statistical analysis was conducted using the SPSS software for Windows, version 22 (originally performed using SPSS software for Windows, version 17; SPSS, Chicago, IL, USA). The threshold for significance was set at *p* ≤ 0.05.

## 3. Result

### 3.1. Higher CRP Levels Are Significantly Associated with Lower Hb Levels in Febrile Children

**Figure 2:**
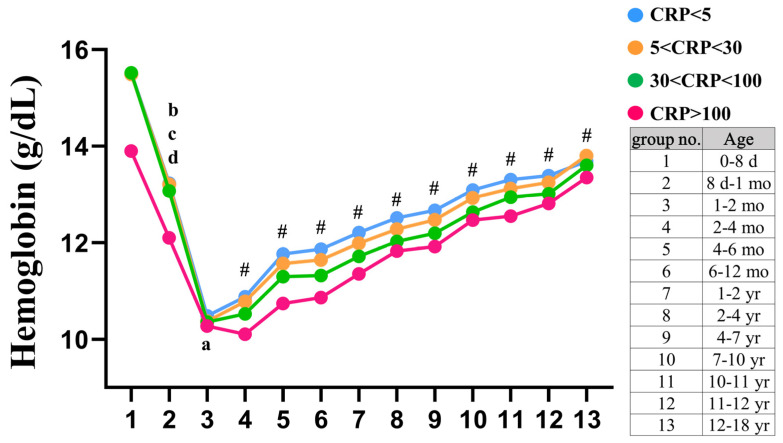
The hemoglobin (Hb) levels vary depending on age, especially in the infant period, and C-reactive protein (CRP) levels. The Hb levels are the highest immediately after birth and subsequently undergo a rapid decline, reaching their lowest point at around 1–2 months of age, and then followed by a steady increment in Hb levels throughout childhood and adolescence. Furthermore, there is a significant association between higher C-reactive protein (CRP) levels and lower Hb levels across all age subgroups. ^a^ *p* < 0.05, CRP ≤ 5 versus 5 < CRP ≤ 30; ^b^ *p* < 0.05, CRP ≤ 5 versus CRP > 100; ^c^ *p* < 0.05, 5 < CRP ≤ 30 versus CRP > 100; ^d^ *p* < 0.05, 30 < CRP ≤ 100 versus CRP > 100; ^#^ *p* < 0.05 between all groups.

The research cohort consisted of 255,019 visits by individuals under the age of 18 during the period from 2010 to 2019. Figure 1 presents a flowchart that illustrates the methodology employed in this study. There were 106,369 febrile children (41.7%) who received laboratory tests. We excluded patients with catastrophic illness (N = 4123) and patients with Hb below 7 g/dL or MCV < 60 pg/cell (N = 3674). After the necessary selection process, a total of 98,572 patients remained eligible and were included in this cohort for further analysis. Hb levels in children are known to display fluctuations that are intricately tied to their age, a phenomenon especially pronounced during the infant phase. Furthermore, these Hb levels can be subject to modulation by the presence of inflammatory disorders, thereby underscoring the complexity of interpreting these measurements in pediatric healthcare. Given the substantial number of children in the study, we categorized the febrile participants into 13 groups to account for potential age-related differences (age: 0–8 d, 8 d–1 mo,1–2 mo, 2–4 mo, 4–6 mo, 6–12 mo,1–2 yr, 2–4 yr, 4–7 yr, 7–10 yr, 10–11 yr, 11–12 yr, and 12–18 yr). Additionally, we further divided them into four groups to address potential variations of CRP based on specific age ranges (CRP levels: <5 mg/L, 5~30 mg/L, 30~100 mg/L, >100 mg/L). The detailed Hb reference values for febrile children are presented in Table 1, Table 2, Table 3 and Table 4. These tables provide detailed information on the specific Hb reference ranges corresponding to different age groups or subgroups within the febrile pediatric population. As depicted in Figure 2, the Hb levels were observed to be highest immediately after birth and subsequently underwent a rapid decline, reaching their lowest point at around 1–2 months of age and then a consistent rise in Hb levels during the developmental stages of childhood and adolescence. This trend indicates a notable wide variation of Hb concentration during the infant period. Furthermore, the study revealed a significant association between higher CRP levels and lower Hb levels across all age subgroups (Figure 2).

### 3.2. Higher CRP Levels Are Significantly Associated with Lower Z Scores of Hb in Febrile Children

**Figure 3:**
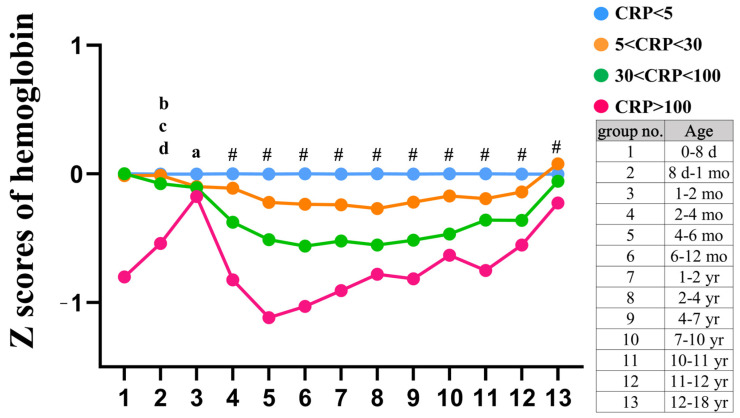
The Z scores of hemoglobin (Hb) vary depending on C-reactive protein (CRP) levels. There is a significant association between higher CRP levels and lower Z scores of Hb across all age subgroups. ^a^ *p* < 0.05, CRP ≤ 5 versus 5 < CRP ≤ 30; ^b^ *p* < 0.05, CRP ≤ 5 versus CRP > 100; ^c^ *p* < 0.05, 5 < CRP ≤ 30 versus CRP > 100; ^d^ *p* < 0.05, 30 < CRP ≤ 100 versus CRP > 100; ^#^ *p* < 0.05 between all groups.

**Figure 4:**
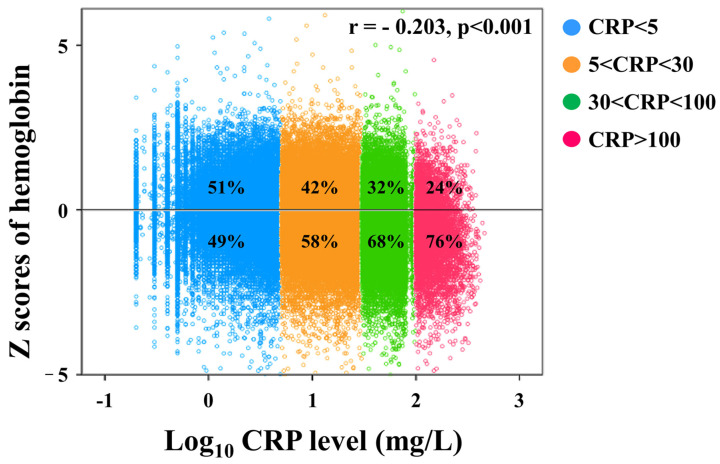
The Z scores of hemoglobin (Hb) vary depending on C-reactive protein (CRP) levels. Acknowledging the wide-ranging distribution of CRP levels, we chose to logarithmically transform CRP for our analytical approach. Of particular significance, our results vividly unveil a discernible trend: the ratio of Z scores for Hb below zero in contrast to those above zero fluctuates from roughly 1:1 in the CRP < 5 mg/L group to approximately 3:1 in the CRP > 100 mg/L group. There was also a significant negative correlation between CRP levels and the Z score of Hb in febrile children (r = −0.203, *p* < 0.001).

**Figure 5:**
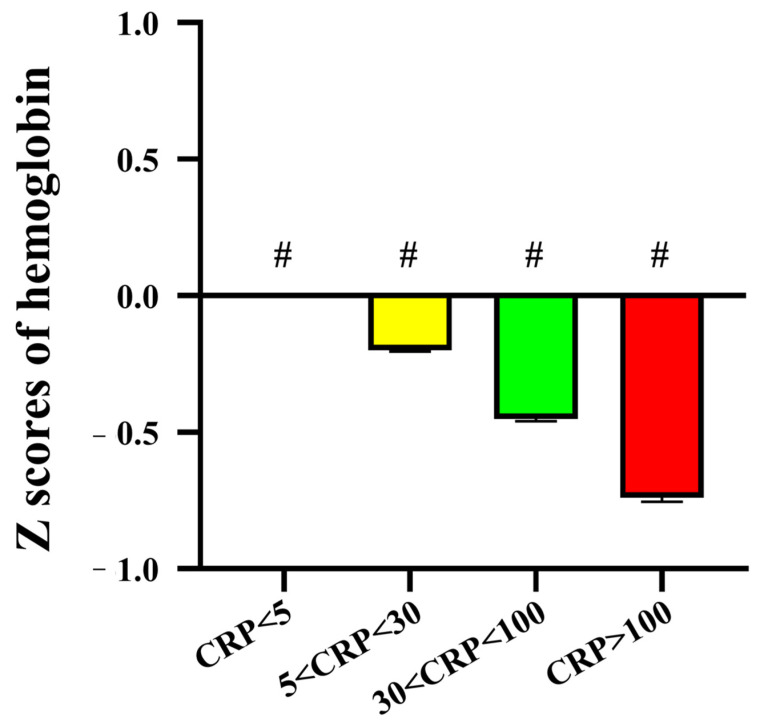
Elevated CRP levels show a significant association with decreased Z scores of hemoglobin in febrile children. The “#” symbol indicates a significant level of *p* < 0.05 when compared to all remaining groups.

Then, we proposed that the change of Hb should be less in febrile children with CRP less than 5 mg/L and we selected this group (N = 33,664) as the reference group for the Z score of Hb for further analysis. As depicted in Figure 3 and Figure 4, they revealed a significant association between higher CRP levels and more negative Z scores of Hb across all age subgroups. Given the broad distribution range of CRP levels, we opted to take the logarithm of CRP for analysis. Importantly, our findings vividly demonstrate a distinct pattern: the ratio of Z scores of Hb below zero compared to those above zero varies from approximately 1:1 in the CRP < 5 mg/L group to about 3:1 in the CRP > 100 mg/L group (Figure 4). Remarkably, a notably stronger negative correlation was observed between CRP levels and the Z score of Hb in febrile children (r = −0.203, *p* < 0.001, Figure 4). Moreover, elevated CRP levels show a significant association with decreased Z scores of Hb in febrile children (all *p* < 0.05, Figure 5).

**Figure 6:**
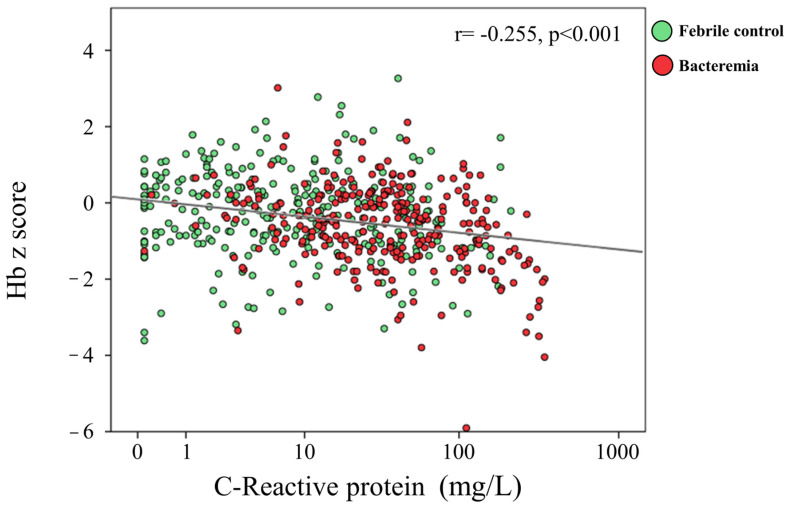
The correlation coefficient between CRP levels and the Z score of hemoglobin (Hb) in children with bacteremia. There is a negative correlation between the Z score of Hb and CRP levels (r = −0.255, *p* < 0.001). In the bacteremia group, there is a notably stronger negative correlation between CRP levels and Z score of Hb (r = −0.41, *p* < 0.001) compared to the non-bacteremia group (r = −0.115, *p* < 0.049).

We have selected true bacteremia cases by the criteria mentioned in the section on blood culture. Among these 98,572 febrile children, there were 291 febrile children who had true bacteremia. To establish a comparison, we also selected a control group consisting of 291 age- and sex-matched febrile individuals of the same period who had negative blood culture results (Table 5). There were higher CRP levels, lower Hb concentrations, and lower Z scores of Hb compared to the non-bacteremia group (Table 5, *p* < 0.001, *p* = 0.003, *p* < 0.001, respectively). As depicted in Figure 6, we observed a negative correlation between the Z score of Hb and CRP levels (r = −0.255, *p* < 0.001). Notably, there was a more significant negative correlation with CRP levels and Z scores of Hb in the bacteremia group (r = −0.41, *p* < 0.001) than in the non-bacteremia group (r = −0.115, *p* < 0.049). This contrastingly substantial correlation highlights the potential clinical significance of CRP as an indicator of Hb variations in the context of bacteremia.

## 4. Discussion

To our knowledge, no reference to Hb levels in febrile children has been proposed. By utilizing a large real-world sample from febrile children, we were able to objectively assess the association between Hb and CRP levels and provide a reference. The present study possesses several notable strengths, and the key findings of this study can be summarized as follows: First, it encompasses a large number of children (N = 98,572), providing robust statistical power and enhancing the reliability of Hb references of their specific age. Second, increased inflammation, as indicated by higher CRP levels, is linked to decreased Hb levels in febrile children, regardless of their specific age. Third, a noteworthy negative correlation was observed between CRP levels and the Z score of Hb. This finding indicates that as CRP levels increase, the Z score of Hb decreases, suggesting a potential inverse relationship between inflammation, especially for true bacteremia cases, and Hb status (as reflected by the Z score) in the study population.

CRP and Hb are both laboratory markers that can provide valuable information about a patient’s health status, particularly in inflammation status and certain medical conditions. While there is no direct correlation between CRP and Hb levels, the association between CRP and Hb levels has been a subject of academic interest and investigation. Ziv-Baran et al. discovered a weak but statistically significant correlation (r = −0.025, *p* < 0.001) between the Hb and CRP levels in a large sample of 16,095 healthy individuals undergoing routine health examination [33]. Santos-Silva et al. found a significant negative correlation between Hb concentrations and the CRP level (r = −0.42, *p* < 0.001). Also, they proposed the use of the ratio of CRP/Hb as a predictor of hospitalization following ER discharge [34]. Increasing evidence indicates that younger patients with bacteremia tend to exhibit lower Hb levels and higher CRP levels [35,36,37]. These findings indicate the presence of inflammation-related anemia, which is a prevalent and significant clinical concern. However, when it comes to the evaluation of febrile children, clinicians tend to allocate relatively less emphasis on the absolute Hb level. For instance, a Hb level of 11 g/L may be considered normal in infants, but it would be considered low in toddlers. Our findings indicated a peak in Hb levels shortly after birth, followed by a rapid decline, reaching their lowest point at approximately 1–2 months of age. This pattern underscores a notable and wide-ranging fluctuation in Hb concentrations during the infant period. By transforming the absolute Hb level into a Z score of Hb, clinicians gain the ability to assess whether it falls within the normal range or indicates an abnormality. Considering the size of the children’s cohort in the study, we classified the febrile participants into 13 groups to accommodate potential variations related to age. This approach allows for a standardized evaluation of Hb levels, considering factors such as age and other relevant characteristics, thereby enabling clinicians to make more accurate judgments regarding the Hb status of the children. In addition, our study proposed that febrile children with CRP levels less than 5 mg/L (N = 33,664) should exhibit a smaller change in Hb compared to truly healthy children. This age-specific reference for the Z score of Hb holds significant potential as a valuable tool for evaluating Hb levels in febrile children and healthy control individuals.

Anemia is frequently observed in a wide range of inflammatory conditions, including infections, inflammatory disorders, and specific types of cancers [38,39,40,41]. In the year 2000, Krause et al. introduced a peptide initially referred to as liver-expressed antimicrobial peptide-1 (LEAP-1), which was subsequently renamed ‘hepcidin’ due to its expression in the liver and antimicrobial properties [42]. In anemia of inflammation, erythropoiesis is iron-restricted by hepcidin-mediated hypoferremia, and erythrocyte production is suppressed by cytokines acting on erythroid progenitors [43]. Hepcidin plays a vital role in regulating iron metabolism and serves as a critical orchestrator in these processes, influencing the availability and distribution of iron in the body and contributing to the complex mechanisms underlying anemia caused by inflammatory conditions [25,44,45]. During infections or inflammation, hepcidin is induced and becomes active by binding to ferroportin, which is an iron exporter found on the absorptive surface of duodenal enterocytes, macrophages, and hepatocytes [46,47]. This interaction leads to the internalization and degradation of ferroportin, subsequently reducing iron export from these cells into the bloodstream [48]. As of now, ferroportin stands as the sole identified mammalian iron exporter, playing a crucial role in facilitating the transport of iron from one cell type to another [26]. Its function is essential for maintaining iron balance within the body, allowing for the efficient movement of iron between different cell types as needed. Consequently, increased levels of hepcidin cause the sequestration of iron within these cells, restricting its accessibility for systemic utilization and thus playing a role in the onset of anemia of inflammation [49]. Furthermore, hepcidin has demonstrated a direct impact on the proliferation and survival of erythroid precursors, as evidenced by its influence on erythroid colony formation [50].

Numerous clinical studies have consistently revealed a strong correlation between sepsis and hepcidin levels [51,52,53]. Sepsis or bacteremia can lead to a decrease in serum iron levels and is associated with elevated levels of hepcidin [54]. Thus, hepcidin has proven to be a valuable biomarker for evaluating the prognosis and outcome of critically ill patients [29,45,55,56]. Michels et al. further demonstrated that inducing hepcidin during pneumonia is crucial in curbing bacterial spread by restricting extracellular iron availability [57]. They proposed that hepcidin agonists could be a valuable therapy for Gram-negative infections in patients with impaired hepcidin production or signaling. Our previous findings consistently demonstrate that plasma hepcidin levels possess a higher predictive value in distinguishing between bacterial and viral infections, specifically in cases of bacterial enteritis and urinary tract infections in febrile children [52]. These findings are consistent with the results of this study. Within the bacteremia group, there is a notably stronger and more pronounced negative correlation between CRP levels and the Z score of Hb (r = −0.41, *p* < 0.001), in contrast to the non-bacteremia group (r = −0.115, *p* < 0.049).

Recently, we further demonstrated that a novel predictor utilizing a combination of the Z score of Hb and plasma hepcidin with a better discriminatory ability for differentiating from WBC, CRP, and Hb between children with Kawasaki disease—an acute multisystem vasculitis syndrome, which mostly affects genetically susceptible infants and children aged less than 5 years old [58]—and other febrile illnesses [30]. This gains particular relevance in the present era, as artificial intelligence technologies are increasingly applied across diverse areas of disease diagnosis, encompassing conditions like sepsis, Kawasaki disease, and more. We hypothesize that the Z score of Hb will serve as a superior biomarker compared to the absolute Hb level itself in the prediction of Kawasaki disease among febrile children. Additionally, anemia based on age constitutes one of the six specific objective laboratory test findings used to assess suspicions of incomplete Kawasaki disease [59], underscoring the significance of the Z score of Hb for the corresponding age in incomplete Kawasaki disease. Moreover, combining the Z score of Hb might be superior to Hb with other clinical markers and may enhance the predictive ability for identifying critical illnesses such as COVID-19 mortality in the ICU [60], sepsis [61], and liver disease [62]. Of course, this synergistic approach has the potential to improve the accuracy and reliability of diagnosing and predicting these severe conditions, allowing for timely intervention and appropriate management strategies.

Our study has its limitations. It is a cross-sectional study; thus, we are unable to conclude the causation relationship between anemia and febrile illness. Due to the absence of accessible laboratory data about the health status of pediatric individuals without any underlying conditions, it became necessary to designate the cohort characterized by CRP levels of 5 mg/L or lower as the comparative reference group for children deemed to be within the realm of normal health. Nonetheless, it is important to acknowledge that this reference group, while serving as a benchmark, might not entirely encapsulate the attributes of a healthy child.

## 5. Conclusions

The study provides an extensive sample size, encompassing a notable number of children. It revealed that Hb reference values change with children’s age and CRP levels. These new reference ranges are notably narrower than earlier reported. The study also presents updated Hb reference values and Z scores for febrile children, alongside an exploration of their connection with CRP. These insights provide a valuable understanding of febrile pediatric patients’ Hb status and illuminate the potential link between Hb levels/Z scores and inflammatory status.

## Figures and Tables

**Table 1:** Characteristics of febrile children (N = 33,664) with CRP ≤ 5 (mg/L).

Age	Male	Female	Hemoglobin (g/dL)	2.5th~97.5th Percentiles (g/dL)
0–8 d	140	536	15.5 ± 2.0	11.6~19.5
8 d–1 mo	377	952	13.2 ± 2.1	9.1~17.3
1–2 mo	740	509	10.5 ± 1.2	8.2~2.8
2–4 mo	1265	2744	10.9 ± 0.9	9.0~12.7
4–6 mo	656	3756	11.8 ± 0.9	10.0~13.6
6–12 mo	3230	2365	11.9 ± 1.0	10.0~13.8
1–2 yr	4373	1428	12.2 ± 0.9	10.4~14.1
2–4 yr	2957	772	12.5 ± 0.9	10.8~14.2
4–7 yr	1879	312	12.7 ± 0.9	10.9~14.5
7–10 yr	931	536	13.1 ± 1.0	11.2~15.0
10–11 yr	394	952	13.3 ± 1.0	11.3~15.3
11–12 yr	326	289	13.4 ± 1.0	11.4~15.4
12–18 yr	1313	1033	13.7 ± 1.5	10.8~16.6

d: Days old; mo: Months old; yr: Years old. Data expressed as mean ± standard deviation.

**Table 2:** Characteristics of febrile children (N = 38,953) with 5 ≤ CRP ≤ 30 (mg/L).

Age	Male	Female	Hemoglobin (g/dL)	2.5th~97.5th Percentiles (g/dL)
0–8 d	24	19	15.5 ± 1.9	11.8~19.1
8 d–1 mo	142	91	13.2 ± 2.0	9.4~17.0
1–2 mo	366	235	10.4 ± 1.1	8.2~12.6
2–4 mo	1155	699	10.8 ± 1.0	8.9~12.7
4–6 mo	860	545	11.6 ± 0.9	9.8~13.3
6–12 mo	3241	2828	11.6 ± 1.0	9.7~13.5
1–2 yr	4959	4122	12.0 ± 0.9	10.2~13.8
2–4 yr	4018	3119	12.3 ± 0.9	10.6~14.0
4–7 yr	2780	2275	12.5 ± 0.9	10.7~14.3
7–10 yr	1268	1043	12.9 ± 1.0	11.0~14.8
10–11 yr	538	473	13.1 ± 0.9	11.3~14.9
11–12 yr	482	357	13.2 ± 1.0	11.3~15.2
12–18 yr	1862	1452	13.8 ± 1.4	11.1~16.5

d: Days old; mo: Months old; yr: Years old. Data expressed as mean ± standard deviation.

**Table 3:** Characteristics of febrile children (N = 20,346) with 30 < CRP ≤ 100 (mg/L).

Age	Male	Female	Hemoglobin (g/dL)	2.5th~97.5th Percentiles (g/dL)
0–8 d	9	8	15.5 ± 1.9	11.7~19.3
8 d–1 mo	57	42	13.1 ± 2.0	9.1~17.1
1–2 mo	227	121	10.4 ± 1.2	8.0~12.7
2–4 mo	654	418	10.5 ± 0.9	8.7~12.3
4–6 mo	537	388	11.3 ± 0.9	9.5~13.1
6–12 mo	1321	1101	11.3 ± 1.0	9.4~13.2
1–2 yr	2073	1741	11.7 ± 0.9	9.9~13.6
2–4 yr	1968	1605	12.0 ± 0.9	10.3~13.8
4–7 yr	1782	1428	12.2 ± 0.9	10.4~14.0
7–10 yr	863	681	12.6 ± 1.0	10.7~14.6
10–11 yr	328	288	12.9 ± 1.0	11.0~14.9
11–12 yr	320	267	13.0 ± 1.0	11.1~14.9
12–18 yr	1163	956	13.6 ± 1.4	10.8~16.4

d: Days old; mo: Months old; yr: Years old. Data expressed as mean ± standard deviation.

**Table 4:** Characteristics of febrile children (N = 5609) CRP >100 (mg/L).

Age	Male	Female	Hemoglobin (g/dL)	2.5th~97.5th Percentiles (g/dL)
0–8 d	1	0	13.9	
8 d–1 mo	11	17	12.1 ± 2.1	8.0~16.2
1–2 mo	50	20	10.3 ± 1.3	7.8~12.7
2–4 mo	145	92	10.1 ± 1.0	8.2~12.0
4–6 mo	176	113	10.7 ± 1.0	8.8~12.7
6–12 mo	400	342	10.9 ± 1.1	8.8~12.9
1–2 yr	437	375	11.4 ± 1.0	9.4~13.3
2–4 yr	414	359	11.8 ± 0.9	10.0~13.6
4–7 yr	524	400	11.9 ± 1.0	10.1~13.8
7–10 yr	255	205	12.5 ± 1.0	10.5~14.4
10–11 yr	118	94	12.6 ± 1.0	10.6~14.5
11–12 yr	94	70	12.8 ± 1.1	10.7~15.0
12–18 yr	491	406	13.4 ± 1.6	10.3~16.4

d: Days old; mo: Months old; yr: Years old. Data expressed as mean ± standard deviation.

**Table 5:** Characteristics of patients with bacteremia (N = 291) and controls (N = 291).

Characteristics	Controls	Bacteremia Patients	*p*-Value
Age (year)	0.9 ± 0.87	0.9 ± 0.83	0.607
Gender (male)	55.0%	60.7%	0.164
WBC (10^3^/μL)	10.9 ± 5.5	11.6 ± 6.3	0.126
CRP (mg/L)	21.7 ± 33.8	60.1 ± 71	<0.001
Hemoglobin (g/dL)	11.5 ± 1.3	11.2 ± 1.4	0.003
Z score of Hb	−0.26 ± 1.1	−0.62 ± 1.1	<0.001

Data expressed as mean ± standard deviation.

## Data Availability

The datasets generated and analyzed during the current study are not publicly available due to strict ethical regulations of information privacy, but are available from the corresponding author Ying-Hsien, Huang on reasonable request.
